# Eco-psychiatry and Environmental Conservation: Study from Sundarban Delta, India

**DOI:** 10.4137/EHI.S935

**Published:** 2008-09-12

**Authors:** Arabinda N. Chowdhury, Ranajit Mondal, Arabinda Brahma, Mrinal K. Biswas

**Affiliations:** 1 Professor and Head; 2 Research Assistants, Institute of Psychiatry, Kolkata, India

**Keywords:** sundarban, human-animal conflict, environment, community mental health, eco-psychiatry

## Abstract

**Aims:**

This study attempts to examine the extent and impact of human-animal conflicts visa-vis psychosocial stressors and mental health of affected people in two villages adjacent to Sundarban Reserve Forest (SRF) in the Gosaba Block, West Bengal, India.

**Methods:**

Door to door household survey for incidents of human-animal conflicts, Focus Group Discussions, In-depth Interviews, Case studies, Community Mental health clinics and participatory observation.

**Results:**

A total of 3084 households covering a population of 16,999 were surveyed. 32.8% people live on forest-based occupation. During the last 15 years 111 persons (male 83, female 28) became victims of animal attacks, viz, Tiger (82%), Crocodile (10.8%) and Shark (7.2%) of which 73.9% died. In 94.5% cases the conflict took place in and around the SRF during livelihood activities. Tracking of 66 widows, resulted from these conflicts, showed that majority of them (51.%) are either disabled or in a very poor health condition, 40.9% are in extreme economic stress and only 10.6% remarried. 1 widow committed suicide and 3 attempted suicide. A total of 178 persons (male 82, female 96) attended the community mental health clinics. Maximum cases were Major Depressive Disorder (14.6%), followed by Somatoform Pain Disorder (14.0%), Post Traumatic Stress Disorder-animal attack related (9.6%) and Adjustment Disorder (9%). 11.2% cases had history of deliberate self-harm attempt, of which 55% used pesticides.

**Conclusions:**

Improvement of quality of life of this deltaic population by appropriate income generation and proper bio-forest management are the key factors to save their life as well as the mangrove environment of the Sundarban region.

## Introduction

**Figure 1.**
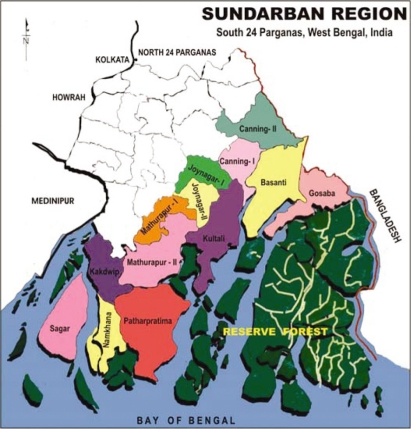
Sundarban under South 24 Parganas district (Not to scale).

Sundarban is the largest intertidal delta in the world and harbours the largest mangrove vegetation. It is bounded by the Hugli river on the West, Ichamoti-Kalindi-Raymongal rivers in the East, Dampare-Hodges line in the North and Bay of Bengal on the South (Fig. 1). The area lies between 21° 30′–22° 45′ North latitude and 88° 66′–89° 5′ East longitude, consisting of a group of 54 islands, innumerable rivers, rivulets, creeks and mangrove forest. It hosts a national park, a tiger reserve and three wildlife sanctuaries.

Indian Sundarban covers an area of 4,262 sq km. Government of India (GOI) constituted Sundarban Biosphere Reserve in 1989 to promote and facilitate conservation and harmony between man and environment. In November 2001, UNESCO recognized Sundarban Reserve Forest (SRF) under its Man and Biosphere programme. Sundarban Tiger Reserve (total area of 2,585 sq km) was created in 23rd December 1973.GOI has identified SRF as Ramsar Site (a wetland of international importance). For its unique biodiversity, UNESCO had declared Sundarban National Park as a World Heritage Site in 1987. Sundarban is the only mangrove forest in the world, which is the home of tigers and having the highest population of tigers in the world.

The Sundarban ecosystem is a unique natural wonder of the world and carries a great ecological significance. It has a rich biological diversity of aquatic and terrestrial flora and fauna and the mangrove forests (1). The Sundarban mangrove forest supports 334 species of plants, 44 species of fishes, 8 species of amphibians, 53 species of reptiles, 315 species of birds, 49 species of mammals (2). Sundarban’s highly productive ecosystem acts as a natural fish nursery; mangrove acts as a natural shield against the fury of cyclonic storm and prevents erosion due to tidal action and checks atmospheric pollution. Finally, millions of people depend on Sundarban ecosystem for their livelihood and sustenance through fishing, collection of honey, fuel wood and timber.

Sundarban is one of the poorest and most densely populated regions of South Asia, with an estimated 8 million people (India and Bangladesh combined) directly dependent on its fragile ecosystem. The Indian Sundarban comprises 19 community development blocks- 13 under South 24 Parganas and 6 under North 24 Parganas district of West Bengal with a total population of 4.1 million (3). 44% of population belongs to schedule caste and tribe, 85% are living on agriculture, of which 90% are landless agricultural labourers and marginal farmers. Other occupations are fishing, pisciculture, woodcutting and honey collection. The level of literacy and per capita income is much below the state average and most of the people fall below the poverty line. The communication and transport network is very poor and most of the areas are inaccessible. Provision of health care is extremely poor and electricity is almost non-existent. Frequent climatic insult is a regular feature—cyclonic storm; inrush of tidal waves and flooding is the cause of recurrent damage of life, crops and property every year. There are 3,500 km man-made embankments on the rivers and water channel around the human settlements to prevent the tidal floods and estuarine saline water from entering the agricultural fields. Sundarban is an extremely backward region with a very poor quality of life of its inhabitants (4).

**Figure 2.**
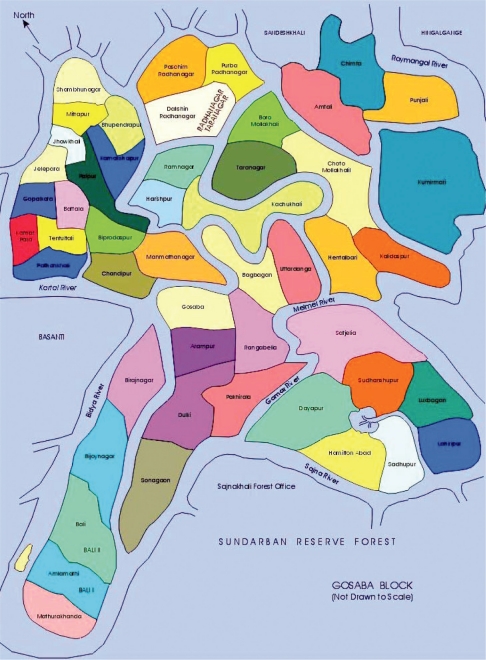
Gosaba Block (Not to scale).

Gosaba is one of the Sundarban blocks under South 24 Pargans, bounded by the *Matala* and *Zilli* rivers/creeks and within the geographical coordinates of 22° 9′ 47″ North, 88° 48′ 10″ East. It is the last inhabited area before the deep forests start. It is about 110 km from Kolkata, the state capital. The total population is 222,764 (male 113,827, female 108,937) (3). It has 14 Gram Panchayat (local self-government unit) of which Bali I and II, Gosaba, Lahiripur and Satjelia Panchayats are facing the SRF, seperated by *Sajina*, *Gomor* and *Melmel* rivers (Fig. 2).

In the context of a rural mental health programme in the Sundarban Delta (5), a community study had previously identified deliberate self-harm by pesticide poisoning and human-animal conflicts as a locally recognized priority problem. This research was thus undertaken with reference to a framework that examined the problem of human-animal conflicts in relation to occupational nature, socio-economic factors and its impact on mental health and environment. This study was conducted from August 2004 to March 2005.

## Materials and Methods

**Figure 3.**
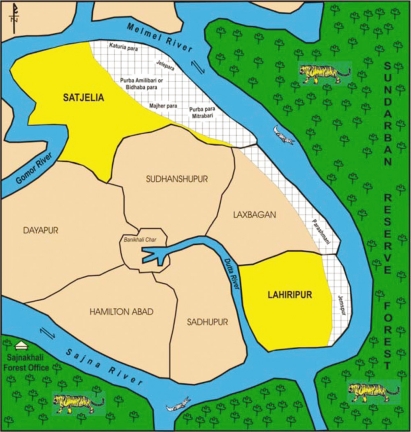
Study area and SRF (Not to Scale).

1. **Study area:** Two villages, Lahiripur and Satjaleia under Gosaba block were selected. The rivers *Melmel* and *Sajina* separate these two villages from the SRF. We selected the hamlets situated on the river-bank because most marginal and poorest people resides here and this belt is most prone to ecological adversity (storm—rain-flood) and animal attack. The hamlets were: Katuriapara, Jalepara, Purba Amlibari, Majher para and Purbapara in Satjelia and Jamespur in Lahiripur (Fig. 3). A total of 3,082 households (Satjelia 1572, Lahiripur 1512), were surveyed.
2. **Survey for human-animal conflicts:** A door-to door survey were done to elicit information on: (1) Number of working adults with a special focus on traditional mode of livelihood, viz. fishing, woodcutting, honey collection, crab collection, Tiger Prawn Seed (TPS) collection and apiary and (2) Number of incidents of any human-animal conflict (tiger, crocodile, shark) in their family during the last fifteen years. Incidents of other animal bites like cat, dog, monkey and snake is reported elsewhere. The information on animal attacks was collected from the survivor or spouse or nearest family member(s) of the deceased. In a few cases, information was also collected from the village Panchayat.
3. **Focus Group Discussion (FGD):** Seven FGDs with different groups of 8–12 persons (victims of animal attacks, villagers, widows of tiger attack victims, hospital staff, forest guard, woodcutters, honey collectors, TPS/crab collectors) was conducted. The themes of the discussions were livelihood measures, context and aftermath of animal attacks and probable solutions.
4. **In-depth Interviews (IDI):** Thirty-two individual interviews from both the villages were taken to elicit their perception about the human-animal conflicts and different aspects of the problem and its probable solution. The persons interviewed were: 3 Crab collectors, 5 TPS collectors, 4 Crocodile attack survivors, 4 Tiger attack survivors, 2 Moulays, 2 Boulays, 6 widows of tiger victims, 2 school teachers, 2 apiary workers, local insurance agent, and a Block medical officer.
  All the In FGD and IDI discussions and narratives were recorded with their consent and transcripted afterwards.
5. **Medical Clinics:** Four mental health clinics, two in each village was conducted on prescheduled dates and Panchayat Pradhan (Head) and Multi-purpose Health Workers of the Block Primary Health Centre were requested to refer persons with mental and nervous problems and those with history of animal attacks. After careful screening 94 from Satjelia and 84 from Lahiripur village were seen and appropriately advised. Psychiatric diagnoses were made following the DSM-IV guidelines (6).

## Results

### Village survey

Table 1 shows the demographic pattern of the study population. 56.5% of total population of Satjelia lives on the riverbank in different hamlets. 36.4% of total population of Lahiripur lives on the bank in a continuous hamlet. Both men and women are involved in different livelihood measures. 34.3% of the adult population in Satjelia and 51.6% in Lahiripur are involved in some economic activities. Though cultivation is the major occupation in Satjelia (42.1%) and Lahiripur (52.2%) but traditional livelihood measures constitute the second most frequent occupation-slightly more in Satjelia (38.9%) than Lahiripur (27.7%). Ratios of male-female participation in all the occupations are more or less equal.

Among the total of 111 cases, 39.6% took place within last 1–5 years, 23.4% between 6–10 years and rest 36.9% before 11 years or more. More than half of the attacks took place during the winter months, viz. November, December and January (53.1%). Table 2a shows the incidents of human-animal conflicts (tiger, crocodile, shark) during the last 15 years from these two villages and at least 111 persons (male 83, female 28) were victimized. Male casualties were higher in Satjelia (63.9%) while that of females was in Lahiripur (57.1%). Occupation of most of the male victims involved deep-forest activity (woodcutting, fishing, honey collection- 69.9%) while in females it was near-forest activities (TPS/Crab collection- 82%). Tiger attack was the commonest (82%) followed by Crocodile (10.8%) and Shark (7.2%). Early part of the morning (55%) and evening (31.5%) was the most prevalent time of attack. Most of the attacks, especially by tigers, took place on land (49.2%) and that of crocodile and shark mostly in the shallow water of the forest canals (28.8%) inside the SRF. Tiger attack was more common when people are engaged in group activities like woodcutting (42.2%) and honey collection (4.8%), which are primarily a male activity and fishing (24.3%). Crab and TPS collection are mainly solitary activity and more females (67.8%) were involved than the males (21.6%). Interestingly, straying of tiger from the forest was the cause of conflict in 4.5% cases. The fatality rate was very high, mostly because of tiger attack −85.5% in males and 39.3% in females. Only 28.8% of the victims had some form of treatment after the attack. Only 10.8% of the survived victims or their family members received some compensation/insurance money.

Table 2b shows the present status of the survived spouses of the victims. Out of 71 male deaths 66 were married at the time of conflict and thus there were 66 widows. 5 widows subsequently died. 10.6% remarried and 51.5% widows are currently suffering from extremely poor health condition and resultant disability. Most of them are in extreme poor economic situation, earning their livelihood either by begging (16.7%), cattle grazing (9.1%) or working as maid servant (15.1%). 2 widows were socially boycotted because of some cultural stigma attached with their tragic life events. One widow, now 43 years old who lost her husband by tiger attack within a month of her marriage and she had a baby boy posthumously. Another widow, now 32 years old also lost her husband and brother-in-law in the same incident of tiger attack shortly after her marriage. The in-laws declared that she had some unholy traits that cause the double accidental death in the family and after a course of severe mental and physical torture drove her out from the family. Out of the 11 female deaths, 7 were married and thus there were 7 widowers, of which 2 died and 3 remarried.

### Major findings from FGDs and IDIs

Following is a brief summary of opinions solely expressed by the participants, in relation to livelihood measures and human-animal conflicts.

#### Agriculture

Agriculture is a hard task in the island mostly because of high salinity of soil and untimely rain and storm. The amount of cultivable land area is gradually decreasing because of new human settlements and division of land property after breaking of joint families. There are no irrigation facilities; the mono crop (paddy) agriculture is totally rain dependent. Unpredictable draught or flood is a frequent cause of crop failure and often intensifies human sufferings. In order to safeguard the best yield of paddy, farmers use, often overuse, different types of pesticides. Pesticides are easily available, even in grocery shops. Pesticides related deliberate self-harm and suicide is a major health issue in the island. At least 15 cases (12 female and 3 male) of deliberate self-harm either by pesticide ingestion (13) or yellow oleander seed ingestion (2) took place from these villages during the last one-year. Many farmers are having pesticide-related illnesses. Pesticide contamination is also causing great damage to the environment. In the paddy field and local ditches all the fishes died, foxes in the bushes also died after eating pesticide poisoned fishes, grasses grow poorly and scanty and the cattle, goats are dwarf and are of weak health.

#### Fishing

Fishing is the second major means of livelihood. Fisherman using the traditional small and slick boat and nylon net explore the big rivers and small creeks within the SRF. During the high tide they spread their nets and squeeze them in low tide. In many occasions they spread the net for 2–3 days. Fishing is usually a group activity, where people work as a rented labour. The proprietor of the boat and net pay them on a day basis. This payment varies with the nature of fishing expedition. In a river trip for a day or two, usual payment per person is Rs. 40 (U.S. $85 cents) per day, for a distance travel over a week it is Rs. 150–180 (U.S. $3–4) per person per day. The fishermen have to enter the rivers and creeks to catch fishes where tiger may hide behind the *Hental* bushes on the bank. Hental bushes are the popular place where tiger can camouflage easily. Even the tiger attacks sleeping fisherman on the boat at night. Forest department records says that during 1975–85, tigers killed 325 fishermen. Sea storm and rain is another danger that often causes loss of life of many fishermen. Every fishing trip on the Sundarban rivers is potentially dangerous for tiger or crocodile attack, so until the fishermen returns, their wives observe some self-punishing rituals like starving the day and taking only the dinner and avoids putting the vermilion dust on their forehead and praying to God every day. Frequently, people take loan to hire a boat and net. There is a wide network of moneylenders and illiterate and simple-natured fishermen are very often exploited through different demands. Recently increasing river-robbery is another threat to the fishermen.

#### Crab collection

Mud crabs are the most lucrative estuarine crabs in Sundarban in terms of its market demand. On average a person catches 10–15 big crabs from the river bank and fetch instant value of Rs. 100–150 (U.S. $2–3). In every island there is a broker in the main market who purchases the day’s yield from the crab collectors. This is a big earning for the poor people of Sundarban. The low mud flat on both sides of forest canals (*Khari*) are potentially best areas to find them and these areas are also the most probable sites for tiger and crocodile attacks. It is primarily a solitary and time consuming activity and mostly housewives and widows are sustaining on this activity. There are many incidents of death and disfigurement due to tiger and crocodile attacks during crab collection.

#### Tiger prawn seed (TPS) collection

TPS collection is the most popular method of earning instant cash in whole of coastal Sundarban. Locally this occupation is called “*Meen-Dhara*” (*meen* means juvenile prawns or seedlings and *dhara* means catch). There is an extensive network of traders and brokers who collect TPS from the river bank and transported to the main market. Depending on the season the rate per TPS varies between Paise 40 to Rs.1.50 and on average a person earns about Rs.10–15 (U.S. $25–30 cents) on the spot. It is a daylong activity and the availability of TPS is dependent on timing of high tide. For its ready cash-reward a vast majority of persons (adult, old, children, housewives, school student) are engaged in this activity. TPS collection near SRF definitely prone the collector to tiger attack and as they are always working in the waist-deep river water, so shark and crocodile attack is also common. Many cases are here who were seriously deformed after such attacks (lost part of the feet, toes or leg) and many were killed. Very recently one such devastating incident of Crocodile attack took place here where the woman was killed during TPS collection but because of the shouting of the villagers, crocodile left her body. The angry villagers put high amount of pesticides in the body of the deceased and placed her near the conflict area and next day the crocodile came and ate the corpse and died consequently. Recently Forest Department and Panchayat are asking not to engage in *meen* collection for its serious influence on coastal environment but this is the only way of earning from the nature for poor people-how one can stop them from earning their bread! It is a free occupation; no license or permission is needed—so everyone is finding it as the only means of earning and living. There is a good demand in the market as well. This activity has been going on for many years and recently the availability of juvenile prawns are decreasing.

#### Wood cutting

The yearly average timber collection from SRF is about 1,20,000 quintals. Forest timber is the most valuable material for construction of houses, boat and other daily necessities of life. Forest department, from time to time, issue a pass for clearing the heavy growth of forest and allow limited number of people to enter the forest. The collected wood is the property of the government and the woodcutters get an amount of money from the bidders. Bidders are the middle traders who hire different woodcutter groups scattered throughout the Sundarban blocks. Each group consist 10–15 people with a leader and a *Boulay. Boulay* is a man with traditional expertise, who knows the magic of keeping the team out of danger in the forest and they also have the supernatural power to make the work area protected so that tiger cannot enter into this chanted territory. They also know the chant by which they “shut” the mouth of the tiger, nick named as “*great uncle*”. *Boulay* don’t take part in woodcutting, they only safely lead the team into the forest like a pilot. As they are the first person to enter the jungle or walk in the front line to do his rituals (like purification of the area, uttering chants to protect everyone and sprinkling of chanted water to the area etc) before the main activity, it is he who was attacked first by the tiger. The tiger killed many *Boulays* and injured many and thus many have left this profession. There is a provision of insurance, called “janata pass” for those who take the pass from the forest department for entering the SRF. But the reality of the fact is that more than 60% of woodcutters in Sundarban are illegal intruders.

#### Fuel wood collection

People from these villages are 100% dependent on forest wood for their household fuel. There is no other types of fuel available in these remote islands. Poor people cannot buy fuel wood from the market. A large number of people are engaged in collecting firewood from the forest almost on daily basis. In addition, different types of wood and leaves are necessary for construction of walls and thatching of the mud huts. Fuel wood collection is done by both solitary and group activity. The groups usually invade the forest with small traditional boats but in solitary activity people explore the riverbank of SRF and collect fuel wood from the vicinity. For group activity there is a provision of getting a ‘pass’ from the Forest department but it involves some payments to the brokers and poor people mostly explore forest illegally be it a small group or single individual. Forest entry is always risky due to tiger attack and many people are killed or injured every year. Many widows are living on this activity. Recently a government plant for bio-gas to produce electricity was installed in Gosaba, but only the rich can afford electricity cost, the poor people are lamenting because the plant engulfs quite a large amount of forest woods daily.

#### Honey collection

Honey and wax collection is a seasonal activity. A small team with a *Moulay* usually does this job. *Moulay* is a especially skilled persons who can identify the beehives in the forest by observing the directions of the flying bees and they also possess supernatural power to sense forest dangers. They lead the team of 4–5 persons in the jungle. During the activity they also protect the area with chants so that tigers cannot enter into this chanted zone. Nowadays because of the danger of tiger attack and also of its seasonal nature, many *Maulays* are giving up this traditional occupation and turned into a day labour or engage in fishing. Recently Forest department issued a ‘pass’ for entering into the forest. Government trip is of 15-days duration and the honey is deposited with the forest department, which in turn pays them a commission. Last year about 15,000 kg honey was collected. The forest department markets the honey. But many people enter the jungle illegally as the price of honey given by government is too low than private traders and secondly there is always some nasty brokers’ play in issuing of license. In a honey collection team women are not allowed. Honey collection is a high-risk activity. As this activity is done solely within the habitat of tiger so it carries a great risk of man-tiger conflict. It is also a very difficult and coordinated task. The steps are: smoking for forced bee-flying, then cutting of the honey-packed part of the hive and honey collection from beneath the tree within few minutes and all are in the midst of the fear of tiger plus the painful sting of giant forest bees. The summer months of March–July is the flowering season of the mangroves and thus of honey collection and this is also the period when the salinity in the rivers reach at its peak and this is also the period when tigers are becoming more man-eater. During the last 10–15 years, tigers had killed more than 75 honey collectors. Recently the government has started an insurance scheme, worth of Rs.50,000 for the honey collector. When the men folk go off for several days in the jungle, their wives spend the days in prayers to the goddess *Bonobibi* (Queen of the forest) for their safe return and in many families, they fast for the whole day, only eating one meal at night and do not take bath or dress themselves until the return of their husband.

#### Human-animal conflict

Human-animal conflict is a part of life here. It is not only when someone is in the forest or near it, but also when at home, may be exposed to danger. There are at least 4 incidents in the last few years when a tiger came to the village and in one occasion killed one villager and in the other, injured the whole family and killed their livestock. Government is saying they should preserve the “badabon” (Mangrove forest) and tigers but they should also preserve the people also! Poor people have no other option than entering the forest for their daily bread. The adjoining areas of *Melmel* and *Gomor* rivers are more dangerous than other parts of human settlement in Sundarban because it is just opposite to the buffer zone of Tiger Reserve. Nowadays tigers are also coming more frequently to the buffer area and thus TPS or crab collection in these rivers, are more dangerous. As most of the forest intruders are illegal so they cannot even visit a government hospital when injured because of ‘police case’. They usually take treatments from the local ‘quacks’. Most unfortunate part of the story is that when some one is dead or injured, for fear of legal harassment, they have to keep it secret. On an average, at least 4–5 persons are killed and 10–12 are injured every year from these villages from animal attacks. The tigers are highly movable animal in the forest because of the daily changing of land markings by high and low tides. So tigers move from one place to other in search of food. Nowadays the availability of food is at stake in the forest as well, so many a times during the paddy-harvesting season, tigers enter the dry and dense paddy fields near the villages. They hide there and target household animals first, and often targets human beings if chance prevails. The incidence of straying of tiger has increased recently. After tiger-attack injury many young persons became disabled because of extreme weakness and shrinkage of the body and also from extreme fear from the attack. They lost their work ability forever. In case of licensed woodcutter’s death due to tiger attack, Government gives one time compensation of Rs. 30,000.

#### Problem with widows

There are hundreds of hapless widows in the villages whose husbands had been mauled and killed by man-eaters. This is a burning problem in all the villages of Sundarban. In each village there is a separate hamlet for widows, locally called “Bidhoba Palli” meaning community of the widows. When the husband is killed by either tiger or crocodile, they become absolutely helpless and are usually mistreated by their in-laws. If the wife is young, their misfortune is terrible-the remarriage rate is very low among the tiger-widows because of social stigma. There is no other opportunity of livings in the villages, so when they are driven out by their in laws, either they fled away from the village towards Kolkata or other big towns and survived by having some day-labour job or in many instances, become the victim of women trafficking. Those who are little older they are plunged into absolute misery of extreme economic hardship and poor health. They usually survive by prawn collection or cattle grazing or begging or working as maidservant in other’s house. They have had very difficult times of bereavement. As most of the forest intruders here are illegal, so they cannot even cry loudly for the fear of being identified by forest guard, they got no scope of performing mortuary rights for their husband for obvious reason and also could not perform widow rituals publicly either for the same fear. It is a terrible emotional trauma for the tiger-widows because in 95% cases the body of the victim can never be recovered. They never get any monetary compensation for this loss also. They are subjected to cultural stigma and thus live in a separate hamlet in each village. Though it is a great social, cultural and human problem here but remains a neglected issue till date.

### Findings from mental health clinics

Predominant diagnoses among the males were: Alcohol dependence 14.6%, MDD (Major Depressive Disorder) 10.9%, Somatoform Pain Disorder and Post-Traumatic Stress Disorder (PTSD)-. 9.8% each and Adjustment Disorder 8.5%. 6.1% males had history of Deliberate Self-harm (DSH) attempt. Among the females, most common three diagnoses were: MDD, Somatoform Conversion Disorder and Somatoform Pain Disorder-each 17.7% followed by PTSD 10.4%. 15.6% of females had history of DSH. Among the total cases the most frequent four diagnoses were MDD 14.6%, Somatoform Pain Disorder 14.0%, PTSD 10.1% and Adjustment Disorder 9%. Detailed clinical analysis of some diagnosis (Table 3) showed the presence of sufficient psychosocial stressor at the background. In Somatoform Pain Disorder, especially among the widowers and widows, presence of psychological factors like economic stress, emotional and physical neglect and comorbid poor physical states like malnutrition, anemia and weakness, makes them more prone to varieties of pain symptoms. In PTSD, frightening memory flash-backs related with the animal attack, mainly of tiger (72.2%) was the commonest. Analysis of past DSH reasons showed that marital conflict (30%) and economic stress (25%) were the major cause. Pesticide ingestion (55.0%) was the most common method chosen.

## Discussion

Two serious questions emerged from this preliminary survey: the extent of human sufferings in relation to ecological specificity of the region and secondly the disastrous impact of eco-degradation by human activity. How to balance both sides? This is the most pertinent question to consider in environmental conservation. Following is a brief discussion addressing the extent of nature-human confrontations and its impact, which may provide some insight for the development of an environmental policy relevant to the local socio-cultural context.

### Population growth and socio-economic backwardness

During post-independent period (1947 onwards) and especially after Bangladesh War in 1972, this entire region has experienced a rapid influx of migrated population. In 1951 the area has population of 11,59,559, by 1991 rose to 32,05,552 and in 2001 census it crossed 40,00,000. Deforestation and new human habitation exerted negative impact on the local economy. Even after 60 years of independence, Sundarban remains an isolated, remote backward region of the state (7). The cumulative effect of all the following socio-political factors, viz. extreme population growth, low income level, lack of industries and employment opportunities, lack of electricity and organized transport, rain-dependent monocrop agriculture, raising of the river bed by man-made embankments, frequent cyclonic insult and inflow of tidal waves, make the people of Sundarban totally dependent on natural resources of the mangrove ecosystem at the risk of their life as well as of nature (8). The increasing population pressure not only pushed back the forest frontier but also competing for the resources with the wild animals and the invariable result is the violent human-animal conflict leading to loss of life on both sides. In fact this is one of the cause of extinction of tigers from the Bali and Java of Indonesia (9)—tigers and people are fighting for the common space (10).

### Impact of woodcutting

Recently the Environment Department of Government of West Bengal raised a concern about the region’s extreme population rise and substantial exploitation of the forest (11). Most of the fringe population used mangrove wood as fuel wood because of its high calorific value (ranges between 4700–5300 cal/kg), especially Hentals (*Phoenix paludosa*). About few thousand people are engaged in collecting firewood from the forests on daily basis. Fisherman also used mangroves wood for fish smoking. Some mangroves are also utilized as fodder for cows and goats. The timber value of mangroves is not very good, yet most of the people used it for construction of huts, poles and carpentry items as also for repairing of country boats. *Phoenix* stems are used for construction of walls and leaves for thatching, *Heritiera* for making doors and windows, besides *Lumnitzera*, *Xylocarpus* and *Avicennia* (12). There is a huge gap in demand and the sustainable supply from the forests. Rapid destruction of mangroves creates a vacuum, as their felling cycle is usually very long, for example, the rotation is 20 years for *Heritiera fomes*, 30 years for *Excoecaria agallocha*, and 40 years for *Ceriops sp.* (13). Mangrove forest is a great economic zone as it acts as the nursery for many brackish water animal species including fishes and provides on an average 500 quintal of honey and 30 quintals of wax every year (14).

### Impact of TPS/crab collection

Brackish water aquaculture is now becoming a highly profitable trade because of its high potential of prawn production, especially of Tiger prawn (*Penaeus monodon*). Shrimp exports constitute 75% of total marine products exported from West Bengal. One report shows that the export value of frozen prawn from West Bengal rose from 2.5 crores in 1973 to 60 crores in 1989 (15). There is also a large demand for tiger prawn seed from Bangladesh, resulting in substantial illegal cross-border trade.

One of the main environmental issues related to the shrimp aquaculture industry is the over collection of prawn seed from the wild (16). TPS collection is one of the greatest threats to the Sundarbans’ mangrove ecology. Thousands of people have turned to collecting tiger prawn seeds for ready cash. They use nylon nets, which are dragged along the riverbanks. In the process of harvesting of prawn seeds, around 50 species of finfish juvenile and 28 species of shellfish juveniles are wasted per net per day, making the activity highly unsustainable (17). One study estimated that prawn seed collectors destroy 181.4 million seeds of fin-and shell-fishes during the months from January to September (18). Another study showed that in order to catch 9586 tiger prawn seeds, collectors destroy approximately 1562862 juveniles of other prawn species, 56000 fishes, 1.9 million crabs, 8000 mollusks and a huge bulk of holoplankters (copepods, chaetognaths, mysids, lucifers etc.) and meroplankters (mega-lopa, alima and anomuran larvae). The destruction of seed of finfish and shellfish has been estimated at a total of 454.6 million during the collection seasons in a year. This results in potentially negative effects on the offshore demersal fishery (19). It has further been assessed that only 0.25%–0.27% of the total biota is being taken care of by the seed collectors for onward transmission to the aquaculture farms. This magnitude of loss of valuable larvae of pelagic biota would lead to severe stock depletion that would obviously hamper the energy transference through the food webs in this marine ecosystem (20). It is estimated (21) that 40,000 fishers get an annual yield of about 540 million seeds of *P monodon* and 10.26 billion other fishes from the dense deltaic mangroves of Sundarban. Indiscriminate seed collection causes a severe depletion of balance betwwen the quantities of seeds produced in the nature and the quantity harvasted leading to loss of estuarine biodiversity (22). Constant dragging of nets along the coast and tidal creeks leads to soil erosion, uprooting the mangrove seedlings and saltmarsh vegetation (like *Ipomea pescarpae*, *Sueda maritima* sp.).

In addition, due to direct and prolonged contact with the seawater, the collectors develop occupational hazards like waterborne diseases, skin infections, reproductive tract disease (in females) and some contiguous diseases. Injuries due to shark (locally named *Cammot*) or crocodyle (*Crcodylus porosus*) bites (disfigurement) are also common. TPS collection near the SRF exposed them to tiger attack also. Social problems related to this activity include children leaving the education system in favour of lucrative earning and many adults spend this ready cash on alcohol consumption. They are also subjected to varieties of economic exploitation by the TPS trade network (23). Many environmental activists think that TPS collection is one of the causes of recent ecological disaster in Sundarban (24).

### Increasing human-animal conflicts

Tiger attacks are rising in Sundarban (25) and causing fatality on both sides. It is a two way process: firstly, the constant forest exploitation by humans disturb the tiger habitat and food availability that force the tigers to stray from the forest and haunt the adjoining villages. Secondly, when humans are available in the forest, tigers compensate the dearth of their food. At the core of the problem there are two basic issues, viz. utter poverty and indiscriminate forest use and the mismanagement of tiger habitat by the authority (poaching of not only tiger but also spotted deer, wild boar, marine turtles, horse shoe crab etc., leading to shortage of tiger food). Habitat encroachment and anthropogenic pressure on the forest is the main cause of increased man-animal conflict in the fringe areas of SRF. Yearly toll of killings by tiger and crocodile is around 300 (including Bangladesh) (26). This is also the reason of increasing man-animal conflicts near all the other national parks in India (27–29).

According to the 2004 census (based on the pugmark method) there were 271 tigers in the Sundarban (2). There are at least 159 poaching incidents took place between 1999–2002 (30). Straying of tigers from the reserved forests into the habitations along the northern and western fringes of Sundarban forest result into death of cattle/human beings as well as tiger. Between 1994–95 and 2001–02, there had been 25-recorded cases of tiger straying, leading to death of 10 tigers and during 2002–04, there had been 16 cases of tiger straying with one tiger getting killed. Recently, two tigers (one pregnant tigress) strayed out of the forest area and entered villages. Both were later captured (31). Death of humans by tiger is largely under-reported because most of the forest intruders are illegal. Forest should be forest in the truest sense, but recent evidences showed that the forests are becoming the place of increasing economic activities of the marginal people. A strong network of corruption, underworld activities and vested mafia interests are active behind all forest resources. Not only in Sundarban, the toll of mortality and morbidity from animal attacks is rising throughout India in recent years (32). The huge human impact of conflicts in terms of injury, disability, disfigurement, widowhood, emotional trauma and utter economic hardship is a burning problem in Sundarban but till now is largely neglected.

Reduction of man-animal conflict to zero-level should be the aim of any environmental conservation programme. The basic geo-economic boundary between human settlement and forest must be clearly delineated so that both can live without confrontation of boundary violation. But unfortunately, if one considers the geo-economic distribution of all the national forests in the developing world, it is evident that a large number of marginal and indigenous people survive on forest resources. So this is not an easy task but different models of activity are being tried, involving social-anthropological input (9) and local community involvement with diverse economic (33) and geopolitical agendas (34–36) with some success.

### Mental health and psycho-social stressors

Very brief mental health clinics showed the presence of an impressive morbidity in this marginal population, many of which have a positive relation with community psycho-social stressors and poor quality of life. There is no provision of any mental health services in these islands and access to the health center is also hindered by cost and transport difficulties. The health system in this remote area largely managed by the ‘quack doctors’ and *Gunin-Ojha’s* net work and needless to say that they continue to harbour very poor physical as well as mental health. Poverty, illiteracy and detachment from outer-world make them more superstitious and dependent on traditional help-seeking, for both physical and mental illness (37). So they believe chants and amulets can cure snakebite, *Gunin-Ojha* can cure mental derangement and the Goddess *Bobobibi* can protect them from tiger attacks. Their deep faith in local traditional health system on the one hand and no alternative modern treatment availability on the other, forced them to live with diseases and disabilities, despondency and helplessness, often at the cost of their lives.

Alcohol addiction is increasing in Sundarban in recent years (38). There is regular supply of country liquor from local “Bhati” (rice-beer manufacturing outlet) as also “IMFL” (India-made foreign liquor) from the nearby towns. The present authors believe that the presence of community stressors are operative behind few of the mental health problems, especially adjustment disorders, somatoform pain disorder, PTSD and DSH (39). The conditions of the widowers and widows are extremely poor both in terms of physical health (comorbid with malnutrition, low general physical condition) and mental state (neglect, abuse, isolation and physical incapacity to earn and economic stress). Among the married women, poverty and continuous economic hardship, husband’s alcoholism and resultant torture (both physical and mental, often involving mother-in-law), often some extra-marital affairs are the main reasons of widespread adjustment problems. Marital conflicts and economic stress was the most common causes of deliberate self-harm of the cases. Easy availability of pesticide made it a popular choice for DSH, which is also noted from other parts of Sundarban (40,41). The issue of pesticide-related DSH/suicide of the region calls for an urgent public health attention (42). Among the PTSD cases, 72% were related to tiger attack. Some of them are almost incapacitated because of extreme anxiety and fear, flashbacks of terrible tiger attack memories and avoidant behaviour. Cases of PTSD related with crocodile or shark attack, are also avoiding river and unable to engage in their TPS or crab collection activities. PTSD has strong negative impact on their earnings. These cases had no access to any medical facility and they are living with their dysfunctional state for many years unattended. It is a matter of great shame that though this area is within 110 km from the state capital, but none of the cases had the opportunity to get mental health treatment ever. This was one of the needs for development of a community mental health programme in Sundarban region (43).

### Eco-psychiatry and environmental conservation

Environmental conservation is an imminent task of the national government for keeping the ecological balance safe and also to save the diversity of Sundarban flora and fauna, especially the mangroves and the tigers. Mangroves are among the world’s most productive ecosystems. They enrich coastal biodiversity, yield commercial forest products, protect coastlines, and support coastal fisheries (44). The Sundarban ecosystem is also essential for human existence, supplying oxygen, absorbing greenhouse gases, regulating weather patterns, protecting cyclones and providing rich sources of fish, wood and honey. The tiger is an integral part of the Sundarban ecosystem and its protection is an urgent ecological duty (45).

In order to have a rational and practical policy and approach we have to understand the undercurrent of a negative ecological cycle operative in Sundarban region. Careful analysis of eco-specificity of Sundarban may provide us some insight about the eco-psychiatric aspect (ecological influence on mental health) of the region (46) including the devastating aftermath of human-animal conflicts. Table 4 shows this cycle of events where the eco-specific livelihood factors enhance the eco-depletion (river and forest exploration) and thus influencing the health of both, people and bioreserve. Of particular interest here is the human-animal conflicts related mortality and morbidity. This vicious cycle is continuous and cumulative and needs to be interrupted with alternative options. Development of a reality based conflict resolution programme in the local socio-cultural context and alternative options of livelihood measures will save the nature as well as humans (47,48).

## Conclusion

People and wildlife combines overarching conflicts of interest for the survival. The present work pointing towards the interrelationship between human-animal conflicts at the background of ecological and sociocultural contexts of Sundarban. Basically the conflicts are largely economic one. So proper planning for environmental conservation needs a holistic approach to solve the local economic interest in terms of locality replacement, land use pattern and empowering local community to mitigate conflict situations. Imparting environmental education is a key factor to influence cultural values and attitudes for maintenance of eco-diversity and for changing mind-set of local people. It is only possible by a strong political will that account ecological value for need-based management of community as well as forest by the Government (49,50). But unfortunately that is not the case always as Jalais (26) very aptly pointed out this dichotomy- “fascination, on the one hand, for the natural aspects of the Sundarbans, but on the other, an unsettling silence on the social and human facet of the region”.

## Figures and Tables

**Table 1.** Demographic features of the study population.

**Demography**	**Satjalia**			**Lahiripur**		
Area in Hector		965.67			851.05	
Cultivation Land (Hector)		625.58			649.84	
Total Population		16,693			20,752	
No. of Study Family		**1572**			**1512**	
***Demographic data:***	**Male**	**Female**	**Total**	**Male**	**Female**	**Total**
Study Population:	4,184	4,059	8,243	3389	3248	6637
Population: (0–6 yrs)	599	594	1193	454	472	926
***Total Adult Worker:***	**2330**	**496**	**2826 (34.3)**	**1985**	**1438**	**3423 (51.6)**
Cultivation	987 (42.4)	202 (40.7)	1189 (42.1)	1058 (53.3)	729 (50.7)	1787 (52.2)
Traditional Work (Fishing/Wood cutting/Honey/Crab/TPS collection)	897 (38.4)	204 (41.1)	1101 (38.9)	531 (26.8)	418 (29.0)	949 (27.7)
Agricultural labour/cattle grazing/livestock raising	402 (17.3)	66 (13.3)	468 (16.6)	348 (17.5)	262 (18.2)	610 (17.8)
Businesses/Service	18 (0.8)	14 (2.8)	32 (1.1)	22 (1.3)	18 (1.3)	40 (1.2)
Quack doctor/Gunin-Ojha/ *Sarpo-Baidya* (Snake-bite healer)/herbal healer	12 (0.5)	2 (0.4)	14 (0.5)	8 (0.6)	4 (0.3)	12 (0.4)
Apiary	14 (0.6)	8 (1.6)	22 (0.8)	18 (1.3)	7 (0.5)	25 (0.7)

Figures in the parenthesis are percentages.

**Table 2a.** Situation analysis of human–animal conflicts among the study population.

**Demography and Animal attack scenario**	**Male**		**Female**		**Total**	
	**n 83**	**%**	**n 28**	**%**	**n 111**	**%**
***Village***						
Lahiripur	30	36.1	16	57.1	46	41.4
Satjalia	53	63.9	12	42.9	65	58.6
***Education:***						
Illiterate	53	63.9	21	75.0	74	66.7
Primary (upto 4th standard)	17	20.5	3	10.7	20	18.0
Secondary (5th standard +)	13	15.6	4	14.3	17	15.3
***Primary Occupation:***						
Wood Collection	27	32.5	1	3.6	28	25.2
Fishing	18	21.7	3	10.7	21	18.9
Honey Collection	13	15.7	–	–	13	11.7
Crab collection	12	13.2	9	32.1	21	18.9
Tiger prawn seed collection	11	14.5	14	50.0	25	22.5
Day Labour	2	2.4	1	3.6	3	2.7
***Offending Animal:***						
Crocodile	2	2.4	10	35.7	12	10.8
Shark	2	2.4	6	21.4	8	7.2
Tiger	79	95.2	12	42.9	91	82.0
***Number of Attacks***:						
Once	81	97.6	26	92.8	107	96.4
Twice	2	2.4	2	7.2	4	3.6
***Time of Attack:***						
Morning (5–11 am)	43	51.8	18	64.3	61	55.0
Noon (11 am–4 pm)	5	6.0	2	7.1	7	6.3
Evening (4–6 pm)	31	37.4	4	14.3	35	31.5
Night (6 pm–5 am)	4	4.8	4	14.3	8	7.2
***Place of Attack:***						
Fishing Boat	8	9.6	3	10.7	11	9.9
Forest	38	45.8	6	21.4	44	39.6
Home	2	2.4	4	14.3	6	5.4
*Khari* (Forest Canal)	30	36.2	2	7.1	32	28.8
River Bank	5	6.0	13	46.4	18	16.2
***Activity during attack:***						
Wood Cutting in SRF	35	42.2	–	–	35	31.5
Fishing within SRF	23	27.7	4	14.3	27	24.3
Crab Collection in SRF	9	10.8	9	32.1	18	16.2
TPS Collection in *Khari*	9	10.8	10	35.7	19	17.1
Honey Collection in SRF	4	4.8	–	–	4	3.6
Fuel Wood Collection in SRF	1	1.2	2	7.1	3	2.7
Sleeping (Home)	2	2.4	2	7.1	4	3.6
Domestic Work	–	–	1	3.6	1	0.9
***Fate of Attack:***						
Death	71	85.5	11	39.3	82	73.9
Survived	12	14.5	17	60.7	29	26.1
***Treatment done:***						
Gosaba BPHC	14	16.8	7	25.0	21	18.9
Private	9	10.8	2	7.1	11	9.9
***Benefit (Money) Received:***	9	10.8	3	10.7	12	10.8

**Table 2b.** Fate of the Spouses of animal attack victims.

**Spouse**	**Male death (n 71) widower**	**Female death (n 11) widower**
Number	66 (%)	7 (%)
Natural Death	5 (7.6)	2 (28.6)
Suicide	1 (1.5)	
Attempted suicide	3 (4.6)	
Remarried	7 (10.6)	3 (42.8)
No trace	3 (4.6)	
Disability/Poor health condition	34 (51.5)	1 (14.3)
Beggar	11 (16.7)	–
Maid servant/day labour	10 (15.1)	2 (28.6)
Cattle grazing	6 (9.1)	1 (14.3)
Social Boycott	2 (3.0)	–

**Table 3.** Clinical and psychosocial analysis of some diagnosis.

**Clinical analysis**	**Male (n 82)**				**Female (n 96)**				**Grand total N 178 (%)**
	**S**	**M**	**W**	**T (%)**	**S**	**M**	**W**	**T (%)**	
**Somatoform Pain Disorder: n**		**5**	**3**	**8 (9.8)**	**1**	**7**	**9**	**17 (17.7)**	**25 (14.0)**
Psychological factor (psychosocial stress/neglect)		4	3*	7 (87.5)*	1	5	9*	15 (88.2)*	22 (88.0)*
Medical factor (Malnutrition/anaemia/weakness)		1	3*	4 (50.0)*		2	9*	11 (64.7)*	15 (60.0)*
**PTSD: n**	**–**	**8**	**–**	**8 (9.8)**		**4**	**6**	**10 (10.4)**	**18 (10.1)**
Tiger attack related		6		6 (75.0)		3	4	7 (70.0)	13 (72.2)
Crocodile attack related						1	2	3 (30.0)	3 (16.6)
Shark attack related		1		1 (12.5)					1 (5.6)
Boat accident in storm		1		1 (12.5)					1 (5.6)
**DSH attempt: n**	**2**	**2**	**1**	**5 (6.1)**	**1**	**9**	**5**	**15 (15.6)**	**20 (11.2)**
*Reasons:*									
Conflict with parent	1			1 (20.0)	1			1 (6.7)	2 (10.0)
Broken love affair	1			1 (20.0)					1 (5.0)
Marital conflict		1		1 (20.0)		5		5 (33.3)	6 (30.0)
Economic stress		1		1 (20.0)		1	3	4 (26.6)	5 (25)
Emotional stress						2	1	3 (20.0)	3 (15.0)
Physical abuse			1	1 (20.0)		1		1 (6.7)	2 (10.0)
Pain of physical illness							1	1 (6.7)	1 (5.0)
*DSH Method:*									
By Hanging			1	1 (20.0)			1	1 (6.7)	2 (10.0)
Pesticide ingestion	1	2		3 (60.0)		5	3	8 (53.3)	11 (55.0)
Household chemical	1			1 (20.0)	1	2		3 (20.0)	4 (20.0)
Yellow oleander seed						2	1	3 (20.0)	3 (15.0)

* Presence of both factors. S, Single; M, Married; T, Total.

**Table 4.** Interrelationship of eco-stress and mental health.

**Agriculture**	**→**	**Traditional Livilihood Measures**	**→**	**Environmental Threat**
Ecological Influences:				
Increasing population and decreasing land		Forest/river based eco-depleting activities:		**4**Damage of mangrove system
High salinity and low production		Fishing		5Damage of Tiger habitat
		Wood Cutting		Overexploitation of forest resources
Rain-dependent mono-crop		Fire wood collection		
Frequent climatic insult-rain, drought, tidal inflow		Tiger Prawn Seed collection		Overexploitation of river resources
		Crab collection		
**1**Frequent crop-failure		3Dependency on natural resources		6Human-animal conflicts
**1**Over-use/wrong-use of pesticides-uncontrolled pesticide market-easy availability of pesticides				
**2**Continued economic stress and poverty				
More people are leaning to Traditional Livelihood Measures for survival →				
↓		↓		↓

**Eco-Psychiatric Manifestations.**

1 Increasing suicide and attempted suicide with pesticide ingestion.

2 Increased interpersonal conflicts and maladjustment- affecting mental health.

3 Enhances supernatural beliefs and influence health seeking behaviour.

4 Reduction in eco-resources-fish, honey, other vegetations, -negative impact on forest based living.

5 Straying of tiger and loss of both-tiger and human life.

6 Human-animal conflicts—human mortality and morbidity/social isolation and sufferings of survivors (widows).
